# The Medical Schools

**Published:** 1899-09-02

**Authors:** 


### The Medical Schools.

### LONDON.

With the object of increasing the utility of the following list of medical schools we have added the postal addresses. For further information application should generally be addressed to "The Dean." It is to be noted that, in addition to those in the following list, there are a large number of other hospitals at which clinical instruction can be obtained, and that at many of these non-school hospitals special arrangements are made for post-graduate study. These are matters, however, which do not concern the commencing student.

### St. Bartholomew's Hospital Medical School,

*West Smithfield, E.C.*

The clinical practice of the hospital now comprises a service of 744 beds, of which 70 are at the convalescent hospital at Swanley.

During last year relief was afforded to 6,405 in-patients and to 144,075 out-patients, besides 1,714 women attended in their confinements at their own homes.

The composition fee is 150 guineas.

The total value of the scholarships and prizes is nearly £900 per annum. Many of these are entrance scholarships, and are of considerable value.

There is a residential college for students within the precincts of the hospital.

A very admirable recreation ground has been acquired at Winchmore Hill, which is easily reached by rail.

### Guy's Hospital Medical School.

*London Bridge, S.E.*

The hospital contains more than 600 beds, and receives over 6,600 in-patients in the year. In the clinical wards, which contain 40 beds, the most interesting medical cases are collected for purposes of clinical teaching. A few beds in the gynæcological ward are to be reserved for cases of difficult labour.

The composition fee is £150 guineas.

The following scholarships, &c., are given :—

*Entrance Scholarships.*—£100, £50, £150, £60, £50.

*Other Prizes, &c.*—A large number of these are given, and there are a considerable number of dresserships and other appointments which are greatly valued.

The club's union is situated at Honor Oak Park. Facing the hospital, and connected with it by a subway, is the college, which provides residence for the house physicians and other residents, as well as for about 50 students. It also accommodates the students' club.

### St. Thomas's Hospital Medical School,

*Albert Embankment, Westminster Bridge, S.E.*

The hospital contains 572 beds, 43 of which belong to St. Thomas's Home.

Standing, as it does, opposite the Houses of Parliament, it occupies one of the finest and most prominent positions in London. About 6,079 in-patients are received in the year.

The composition fee is £150 on entrance, or £157 10s. by instalments.

The following scholarships, &c., are given :—

*Entrance Scholarships.*—£150, £60 (with a consolation prize of £20 at the discretion of the School Committee), £50 for those coming up from the Universities.

*Other Scholarships, &c.*—Scholarships and prizes amounting to a value of £300 are awarded at the sessional examinations, as well as several medals.

Many structural improvements have recently been made both in the hospital and in the school buildings.

There is no residential college, but there is a good club. A recreation-ground of more than nine acres is provided at Chiswick.

King's College Medical Faculty,

*Strand, W.C.*

The hospital, which is situated in Portugal Street, Lincoln's Inn, W.C., about five minutes' walk from the college, contains 220 beds, and receives nearly 3,000 in-patients in the course of the year.

The composition fee is £135.

The following scholarships, &c., are given:—

*Entrance Scholarships.*—£75, £75, £60, £40, £20, £60, £40, and one of 70 guineas, and another of 60 for students from Oxford, Cambridge, or other British university.

Other prizes are open to matriculated students which, in the course of the curriculum, amount to £300.

The athletic club has a recreation ground at Wormwood Scrubs.

Residential chambers are provided for a certain number of students.

University College Medical Faculty,

*Gower Street, W.C.*

The hospital contains 185 beds, and the number of in-patients has been about 3,020 in the year. The building is at present undergoing reconstruction, a very large sum having been given by Sir Blundell Maple for the purpose, and the works have, so far, proceeded with such rapidity that it is fair to hope that by the time students now entering have passed their anatomy, and got into the wards, they will have the run of a very fine hospital indeed.

The composition fee is £141 15s. in one sum, or £147 in three annual instalments.

The following scholarships, &c., are given:—

*Entrance Scholarships.*—One of £120 and two of 55 guineas.

*Other Scholarships, Prizes, &c.*, amounting to about £800, are awarded annually.

Charing Cross Hospital Medical School,

*Charing Cross, W.C.*

The hospital contains 180 beds, and is now being extended; and the adjoining Royal Westminster Ophthalmic Hospital, to which Charing Cross Hospital students are admitted, contains 30 beds for eye cases.

The composition fee is £115 10s. on entry, or £127 1s. by instalments. Sons of medical men pay £105, or £115 10s. by instalments.

The following scholarships and prizes are given:—

*Entrance Scholarships* of the value of 100 guineas, 60 guineas, 40 guineas, 55 guineas (sons of medical men), and 30 guineas (dental). One Epsom Scholarship, giving a free education, is placed at the disposal of the Royal Medical Benevolent College, to be competed for by foundation scholars who have passed the preliminary scientific examination for the London M.B. Two Universities Scholarships of the value of 60 guineas each to students of Oxford, Cambridge, and London, who have passed certain examinations, but have not entered any London Medical School.

*Other Scholarships and Prizes* of the value of over £550 are awarded annually.

The athletic ground of the Students' Club is situated at Eltham, and can be reached in half an hour from Charing Cross.

St. George's Hospital Medical School,

*Hyde Park Corner, S.W.*

The hospital contains 351 beds, and about 4,172 in-patients are received in the year.

The composition fee is £150 in one payment, or £160 by instalments.

The following scholarship prizes, &c., are given:—

*Entrance Scholarships.*—£150 (to sons of medical men), £50, £50, two of £85 (for Oxford or Cambridge men, £85 (for students from provincial university colleges).

*Other Scholarships and Prizes* are given of the following value: £100, £40, £30, £32, £32, £18, six of £10 10s. each, and various others.

London Hospital Medical School,

*Mile End, E.*

The hospital contains nearly 800 beds, this being the largest general hospital in the kingdom, and received as many as 11,622 in-patients during the past year.

Being situated near the docks and in the midst of a large manufacturing district, it offers to students an unrivalled field for clinical study. Among the advantages connected with "the London" as a place for study is that living in the vicinity of the hospital is less expensive than in the case of some other hospitals, and that a very large number of resident appointments are open to the students.

The following scholarships, &c., are given:—

*Entrance Scholarships.*—£120, £60, £60, £35, £126, £30, and £20.

*Other Scholarships, &c.*—A large number of scholarships, &c., are given, varying from £10 to £60, and many appointments which practically are to be classed among the most valuable kind of prizes.

The new school buildings, which we mentioned last year, are now completed, so that the work is carried on among very favourable surroundings.

The clubs union athletic ground is within easy reach of the hospital.

St. Mary's Hospital Medical School,

*Paddington, W.*

The hospital contains 281 beds, but when the new buildings are completed 100 more beds will be opened. About 3,961 in-patients per annum are received.

The composition fee is £139 in one payment, or £144 in four annual instalments.

The following scholarships, &c., are given:—

*Entrance Scholarships.*—One of £144; two of 75 guineas; one of 50 guineas; two of 55 guineas, for students from Oxford and Cambridge; one of £144, open to students from Epsom College who are sons of medical men.

*Other Scholarships.*—Four of the value of £20 are given at the end of each summer session.

There is a residential college in connexion with the medical school.

Middlesex Hospital Medical School,

*Berners Street, W.*

The hospital contains 320 beds, of which 35 are specially set apart for cases of cancer. About 3,600 in-patients are received during the year.

The composition fee is 135 guineas.

The following scholarships, &c., are given :—

*Entrance Scholarships.*—One of £100 and one of £60 ; one of £60, which is open to students from Oxford or Cambridge ; and one to students of Epsom College.

*Other Scholarships, &c.*—The Broderip scholarship of £60 and £40, and various prizes. There is a residential college attached to the school and hospital.

Westminster Hospital Medical School,
*Caxton Street, S.W.*

The hospital contains 212 beds. About 2,696 in-patients are received in the year.

The composition fee is 110 guineas on entrance, or 120 guineas in two instalments, or 135 guineas in six instalments.

*Scholarships, &c.*, are given during the year of the following value: £60, £40, £30, £60, £40, £40, £60, £40, £50, £50, £60, £40, £40, £60, £40, a free presentation open to pupils of Epsom College, and a Scholarship in Arts of £20 for dental students.

London School of Medicine for Women,
30, *Handel Street, Brunswick Square, W.*

The number of students at the beginning of last year was 190.

The Royal Free Hospital, in connection with which this medical school is carried on, contains 170 beds. About 2,070 in-patients are received in the year.

Students can also attend the fever hospitals of the Metropolitan Asylums Board, and are admitted to the practice of a considerable number of special hospitals.

The composition fee is £125 in one payment, or £135 in four instalments.

The following scholarships, &c., are given :—

*Entrance Scholarships.*—£30. Bostock Scholarship, £60 a year for four years.

*Other Scholarships and Prizes* are of the following value: £30, three of £20 each, £50, and many others. Some of these, however, are coupled with the condition that the recipient shall practise in India.

Both school and hospital have recently been much improved.

### PROVINCIAL MEDICAL SCHOOLS.

In all the provincial schools the curriculum is so arranged as to give the students the same opportunities of obtaining degrees and diplomas as are open to those studying in London, and certain of the provincial schools, such as those of Newcastle-upon-Tyne and the colleges connected with the Victoria University, have the additional advantage of being connected with universities from which medical degrees can be obtained. What the newly-reconstituted University of London will do for the London students is as yet uncertain; but there can be no doubt that, as things are constituted at present, the hope of obtaining a degree at less expenditure of time and labour than has hitherto been required by the London University is one of the inducements to study at some of the provincial schools, and especially the Scottish schools, which are directly connected with universities. Another thing that has to be borne in mind is that all the provincial medical schools are faculties or departments of large colleges, or, as at Cambridge, are schools connected with universities. They are not mere appendages to hospitals, as is often the case in London, and thus it happens that the fees for hospital practice are in many cases given separately from those for the college course.

Cambridge University.

It is not as a complete medical school that Cambridge occupies the high position it now holds, but as a great science school in which the first half of the curriculum can be passed. The advantages of Cambridge are very great. First, there is the collegiate life, the place of which cannot be taken by any other agency ; next there are the facilities for study on a purely scientific basis which are unsurpassed; then there is the great advantage of the degree which is obtained at the end of the course; and, finally, is to be mentioned the advantage which it is to the student to hail from Cambridge. The student who transfers himself from one school to another in the middle of his course can hardly avoid feeling somewhat of a homeless wanderer. But in the case of the Cambridge student there is a reason for his wandering, and most of the London schools make special arrangements for his reception. In connection with the University course, hospital practice can be followed at the Addenbrooke's Hospital, which has 158 beds, and receives over 1,300 in-patients per annum.

Owens College (Medical Department),
Manchester.

This is one of the colleges of the Victoria University.

The Royal Infirmary in which clinical instruction is given contains 300 beds. Associated with it are a large convalescent hospital and the Royal Lunatic Asylum.

There are many entrance scholarships.

The composition fee is £70 at the college, and £42 for the hospital practice.

A new hospital is to be erected shortly if the arrangements for the sale of the present infirmary site should be satisfactorily completed.

The Owens College is very well equipped for scientific study.

University College (Medical Faculty), Liverpool.

This is one of the colleges of the Victoria University, and it possesses some very fine and well-equipped laboratories which have recently been opened.

The Royal Infirmary, which adjoins the school, contains 300 beds, and various other institutions are open to students.

The composition fee for the "conjoint" is £70 for the college, and 40 guineas for the Royal Infirmary.

The Yorkshire College (Medical Department), Leeds.

This is one of the colleges of the Victoria University.

The Leeds General Infirmary has over 400 beds, and other institutions are also utilised for clinical instruction.

The composition fee for the college is 64 guineas, and for the hospital, &c., 46 guineas.

University of Durham College of Medicine,
Newcastle-upon-Tyne.

The Royal Infirmary, where the clinical instruction is given, contains 280 beds, but a new and larger building is about to be constructed.

The composition fee for the college is 70 guineas, and for the hospital 25 guineas.

Residence at Newcastle for one year out of the five is necessary for graduation at the Durham University.

Mason University College (Queen's Faculty of Medicine), Birmingham.

The General Hospital and the Queen's Hospital, in both of which clinical instruction is given, together contain over 400 beds, and receive 6,000 in-patients in the year.

The composition college fee is £70 on entrance, or in two annual instalments; that for the hospital practice is £42.

Students have also free admission to the City Lunatic Asylum and Fever Hospital, and several special hospitals.

University College (Faculty of Medicine), Bristol.

It is now arranged that students of the college shall be admitted to the clinical practice of the Bristol Royal Infirmary and the Bristol General Hospital conjointly. These institutions contain between them a total of 470 beds, with extensive out-patient and special departments.

The composition fee is 65 guineas for the college course, and 35 guineas for the hospital practice.

University College (Department of Medicine), Sheffield.

Clinical instruction is given in medicine and surgery at the Sheffield Royal Infirmary, which contains 240 beds, and the Royal Hospital, containing 125 beds, and students are also admitted to the Jessop Hospital for Diseases of Women, the City Fever Hospitals, and the South Yorkshire Asylum.

Composition fee for hospital practice £45, and for lectures and practical courses 60 guineas.

University College (School of Medicine), Cardiff.

This school provides instruction for the first three years of the medical curriculum, and prepares for the preliminary scientific and intermediate examinations of the University of London, and corresponding examinations of other universities.

The composition fee for London University students is £57 10s., and for students preparing for the first and second examinations of the conjoint board, £40.

### SCOTTISH MEDICAL SCHOOLS.

Edinburgh University.

School of Medicine of the Royal Colleges, Edinburgh.

Medical College for Women, Edinburgh.

Glasgow University.

Queen Margaret College, Glasgow (the Women's Department of the University).

St. Mungo's College, Glasgow.

Anderson's Medical College, Glasgow.

University of Aberdeen.

University College, Dundee. Connected with the University of St. Andrew's.

### IRISH MEDICAL SCHOOLS.

The School of Physic in Ireland, Dublin.

The Catholic University Medical School, Dublin.

The Schools of Surgery of the Royal Colleges of Surgeons in Ireland, Dublin.

Queen's College, Belfast.

Queen's College, Cork.

